# Gut microbiota dysbiosis characterized by abnormal elevation of *Lactobacillus* in patients with immune-mediated necrotizing myopathy

**DOI:** 10.3389/fcimb.2023.1243512

**Published:** 2023-08-25

**Authors:** Xiuping Liang, Yanhong Li, Lu Cheng, Yinlan Wu, Tong Wu, Ji Wen, Deying Huang, Zehui Liao, Chunyu Tan, Yubin Luo, Yi Liu

**Affiliations:** ^1^ Department of Rheumatology and Immunology, West China Hospital, Sichuan University, Chengdu, China; ^2^ Rare Diseases Center, West China Hospital, Sichuan University, Chengdu, China; ^3^ Institute of Immunology and Inflammation, Frontiers Science Center for Disease-related Molecular Network, West China Hospital, Chengdu, China; ^4^ Department of Rheumatolopy and Immunolopy, Meishan People’s Hospital, Meishan, Sichuan, China

**Keywords:** gut microbiota, immune-mediated necrotizing myopathy, dysbiosis, clinical indicators, lactobacillus

## Abstract

**Aim:**

The gut microbiota plays an important role in human health. In this study, we aimed to investigate whether and how gut microbiota communities are altered in patients with immune-mediated necrotizing myopathy (IMNM) and provide new ideas to further explore the pathogenesis of IMNM or screen for its clinical therapeutic targets in the future.

**Methods:**

The gut microbiota collected from 19 IMNM patients and 23 healthy controls (HCs) were examined by using 16S rRNA gene sequencing. Alpha and beta-diversity analyses were applied to examine the bacterial diversity and community structure. Welch’s t test was performed to identify the significantly abundant taxa of bacteria between the two groups. Spearman correlation analysis was performed to analyze the correlation between gut microbiota and clinical indicators. A receiver operator characteristic (ROC) curve was used to reflect the sensitivity and specificity of microbial biomarker prediction of IMNM disease. P < 0.05 was considered statistically significant.

**Results:**

Nineteen IMNM patients and 23 HCs were included in the analysis. Among IMNM patients, 94.74% (18/19) of them used glucocorticoids, while 57.89% (11/19) of them used disease-modifying antirheumatic drugs (DMARDs), and the disease was accessed by MITAX (18.26 ± 8.62) and MYOACT (20.68 ± 8.65) scores. Participants in the groups were matched for gender and age. The diversity of the gut microbiota of IMNM patients differed and decreased compared to that of HCs (Chao1, Shannon, and Simpson indexes: p < 0.05). In IMNM patients, the relative abundances of Bacteroides, Roseburia, and Coprococcus were decreased, while that of Lactobacillus and Streptococcus were relatively increased. Furthermore, in IMNM patients, Lactobacillus was positively correlated with the levels of anti-signal recognition particle (SRP) antibodies, anti-Ro52 antibodies, and erythrocyte sedimentation rate (ESR), while Streptococcus was positively correlated with anti-3-hydroxy-3-methylglutaryl-coenzyme A reductase (HMGCR) antibodies and C-reactive protein (CRP). Roseburia was negatively correlated with myoglobin (MYO), cardiac troponin T (cTnT), ESR, CRP, and the occurrence of interstitial lung disease (ILD). Bacteroides was negatively correlated with ESR and CRP, and Coprococcus was negatively correlated with ESR. Finally, the prediction model was built using the top five differential genera, which was verified using a ROC curve (area under the curve (AUC): 87%, 95% confidence interval: 73%–100%).

**Conclusion:**

We observed a characteristic compositional change in the gut microbiota with an abnormal elevation of Lactobacillus in IMNM patients, which was accompanied by changes in clinical indicators. This suggests that gut microbiota dysbiosis occurs in IMNM patients and is correlated with systemic autoimmune features.

## 1 Introduction

Immune-mediated necrotizing myopathy (IMNM) is a type of idiopathic inflammatory myopathy (IIM), a group of autoimmune myopathies characterized by severe proximal muscle involvement and necrosis of muscle fibers, which are seen on a muscle biopsy (Miller et al., 2002; Senécal et al., 2017). IMNM includes anti-signal recognition particle (SRP) antibody myopathy, anti-3-hydroxy-3-methylglutaryl-coenzyme A reductase (HMGCR) myopathy, and autoantibody-negative IMNM (Allenbach et al., 2018). Previous studies found that IMNM usually progresses rapidly (Allenbach et al., 2016), mainly manifesting as myalgia and myasthenia, and proximal muscles of the lower limbs are the earliest and most seriously involved. Some patients also have dysphagia, muscle atrophy, and elevated serum creatine kinase (CK) levels (Allenbach et al., 2014; Watanabe et al., 2016; Mariampillai et al., 2018). At present, corticosteroid was the first line treatment of IMNM. Meanwhile, disease-modifying antirheumatic drugs (DMARDs) like methotrexate and ciclosporin, immunoglobulin and rituximab are also the main treatments. However, patients still face the dilemma of poor prognosis without symptomatic relief (Allenbach et al., 2018; Allenbach et al., 2020) An in-depth understanding of IMNM pathogenesis guides the development and use of drugs. Research has indicated that anti-HMGCR and anti-SRP autoantibodies play a pathogenic role and may directly cause muscle damage. Additionally, the activation of classical complement and the infiltration of inflammatory cells, particularly macrophages, are also involved in the occurrence and progression of IMNM (Allenbach et al., 2020; Chen et al., 2023). However, the specific mechanisms behind these processes remain unclear.

The term ‘microbiota’ refers to microbial flora, representing microorganisms that are symbiotic and pathogenic for humans (Adak and Khan, 2019). The gut microbiota participates in various life activities (Ahmed et al., 2016; Gensollen et al., 2016; Blander et al., 2017), but with dysbiosis of bacterial function and diversity, the imbalance of gut microbiota may lead to or contribute to the occurrence of diseases (Hevia et al., 2014; Shimizu et al., 2018). In recent years, several studies demonstrated that gut microbiota might be one of the most important contributors to the pathogenesis of autoimmune diseases, such as rheumatoid arthritis (RA), systemic lupus erythematosus (SLE), antiphospholipid syndrome (APS), and Sjögren’s syndrome (SS) (Hevia et al., 2014; Kamei et al., 2017; Chen et al., 2021; Xiang et al., 2021; Wang et al., 2022; Zhao et al., 2022). The study conducted by Luo et al. (Luo et al., 2022) discovered that there is an imbalance in the gut microbiota of patients with myositis, including 9 dermatomyositis patients, 5 antisynthetase syndrome patients, and 6 IMNM patients. This dysbiosis is accompanied by alterations in inflammatory factors, oxidative stress indexes, and small molecule metabolites in serum. Similarly, Zhufeng et al. (Zhufeng et al., 2022) revealed that the diversity of gut microbiota in individuals with IIM is significantly reduced when compared to healthy controls. Additionally, they noticed an increase in bacteria associated with inflammation, while the levels of certain butyrate-producing bacteria declined significantly. Moreover, it has been reported that the administration of fecal microbiota transplantation or microecological regulators has the potential to treat autoimmune diseases by modulating the immune system’s functionality (Huang et al., 2022; Widhani et al., 2022; Sophocleous et al., 2023; Wu et al., 2023). IL-2 has shown promising results in the treatment of IIM by regulating T cells. And then an intriguing observation has emerged, indicating that IL-2 therapy for IIM may be capable of rectifying the dysbiosis of gut microbiota (Zhufeng et al., 2022). The evidence presented implies that the dysregulation of the microbiota plays a role in the development of IIM, highlighting the potential of manipulating the microbiota as a novel therapeutic strategy. However, in terms of IMNM, which is a subtype of IIM, the presence of dysbiosis in the flora remains uncertain.

In our study, we detected and analyzed changes in the gut microbiota and their correlation with clinical features in the feces of IMNM patients. We hope to offer a novel perspective for exploring the pathogenesis of IMNM or developing therapeutic targets.

## 2 Materials and methods

### 2.1 Participants and sample collection

We registered IMNM patients admitted to West China Hospital of Sichuan University from May 2020 to November 2022 after classification according to the pathological criteria described by the 224th European Neuromuscular Center International Workshop (Allenbach et al., 2018). Then, we excluded individuals with the following: (1) current extreme diets, including binge eating, dieting, spicy diet, or extremely picky eating; (2) major organ dysfunction; (3) serious infection that requires antibiotic, antivirals, or antifungal treatment; (4) malignancy; (5) other rheumatic or autoimmune diseases; (6) the age over 18 years; (7) a history of receiving antibiotics, antivirals, or antifungals within the last three months. Meanwhile, age-and gender-matched healthy controls (HCs) without a personal history of muscular diseases were recruited, who also were family members of hospitalized patients. Ultimately, we enrolled 19 IMNM patients who met the inclusion and exclusion criteria and 23 HCs (**Supplementary Figure 1**). The study procedure was approved by the Biomedical Research Ethics Committee, West China Hospital of Sichuan University (no. 695 in 2020), and written consent was obtained from all participants according to the Declaration of Helsinki.

Fecal samples of all participants, including IMNM patients and HCs, were collected at the Department of Rheumatology and Immunology in West China Hospital of Sichuan University under supervision by a single research coordinator to ensure uniformity of sample collection. Meanwhile, demographic characteristics, clinical features, and laboratory data of INMNM patients at the time of sampling were also collected. These data included age, body mass index (BMI), sex, disease course, complication rates, myasthenia, myalgia, amyotrophy, MITAX score, MYOACT score, medications, antinuclear antibody (ANA), anti-SSA antibody, anti-Ro52 antibody, anti-HMGCR antibody, anti-SRP antibody, aspartate aminotransferase (AST), alanine aminotransferase (ALT), CK, lactate dehydrogenase (LDH), hydroxybutyrate dehydrogenase (HBDH), myoglobin protein (MYO), creatine kinase muscle and brain isoenzyme (CKMB), cardiac troponin T (cTnT), erythrocyte sedimentation rate (ESR), and C-reactive protein (CRP), complement component 3 (C3) and C4.

### 2.2 Microbial DNA extraction and PCR amplification

We used the E.Z.N.A.^®^ soil DNA Kit (Omega Bio-Tek, Norcross, GA, US) to extract total microbial genomic DNA from fecal samples following the manufacturer’s instructions, verified the quality and concentration of DNA by 1.0% agarose gel electrophoresis and a NanoDrop^®^ ND-2000 spectrophotometer (Thermo Scientific Inc., USA), respectively, and kept them at a −80°C refrigerator for later use. The hypervariable region V3–V4 of bacterial 16S rRNA gene was amplified using primer pairs 338F (5’-ACTCCTACGGGAGGCAGCAG-3’) and 806R (5’-GGACTACHVGGGTWTCT-AAT-3’) by an ABI GeneAmp^®^ 9700 PCR thermocycler (ABI, CA, USA). The polymerase chain reaction (PCR) product was extracted from a 2% agarose gel, purified using the AxyPre DNA Gel Extraction Kit (Axygen Biosciences, Union City, CA, USA) according to the manufacturer’s instructions, and quantified using a Quantus™ Fluorometer (Promega, USA).

### 2.3 Illumina MiSeq sequencing and data processing

We pooled purified amplicons in equimolar amounts and paired-end sequenced on an Illumina MiSeq PE300 platform (Illumina, San Diego, USA) according to the standard protocols by Majorbio Bio-Pharm Technology Co. Ltd. (Shanghai, China). Raw sequencing data were demultiplexed using an in-house Perl script, quality-filtered by Fastq version 0.19.6, and merged by FLASH version 1.2.1. Then, the optimized sequences were clustered into operational taxonomic units (OTUs) using UPARSE 7.1 with a 97% sequence similarity level. The most abundant sequence for each OTU was selected as a representative sequence. The number of 16S rRNA gene sequences from each sample was flattened according to the minimum number of sample sequences, which yielded an average coverage of 99%, to minimize the effects of sequencing depth on alpha and beta-diversity measures. The taxonomy of each OTU representative sequence was analyzed by RDP Classifier version 2.11 against the 16S rRNA gene database Silva v138 using a confidence threshold of 0.7. The fastq files were deposited into the NCBI Sequence Read Archive database (accession number: PRJNA937002).

### 2.4 Statistical analysis

Bioinformatic analysis of the gut microbiota was performed using the Majorbio Cloud platform (https://cloud.majorbio.com). Based on the OTU information, rarefaction curves and alpha diversity indexes, including observed OTUs, Chao1 richness, Shannon index, and Simpson index, were calculated with Mothur v1.30.2. We assessed the similarity among microbial communities in different samples by principal coordinate analysis (PCoA) based on Bray-Curtis dissimilarity and ANOSIM using vegan v2.5-3 package. Welch’s t test was performed to identify significantly abundant taxa (phylum and genera) of bacteria between the two groups. The linear discriminant analysis (LDA) effect size (LefSe) analysis was applied to detect the most discriminatory taxa among groups. Different features with an LDA score cutoff of 4.0 were identified. Spearman correlation analysis was performed to analyze the correlation between the gut microbiota and clinical indicators and the correlation network between genera. Finally, a ROC curve was used to reflect the sensitivity and specificity of microbial biomarker prediction of disease. P < 0.05 was considered statistically significant.

## 3 Results

### 3.1 IMNM patients showed dysbiosis in gut microbiota

**Figure 1:**
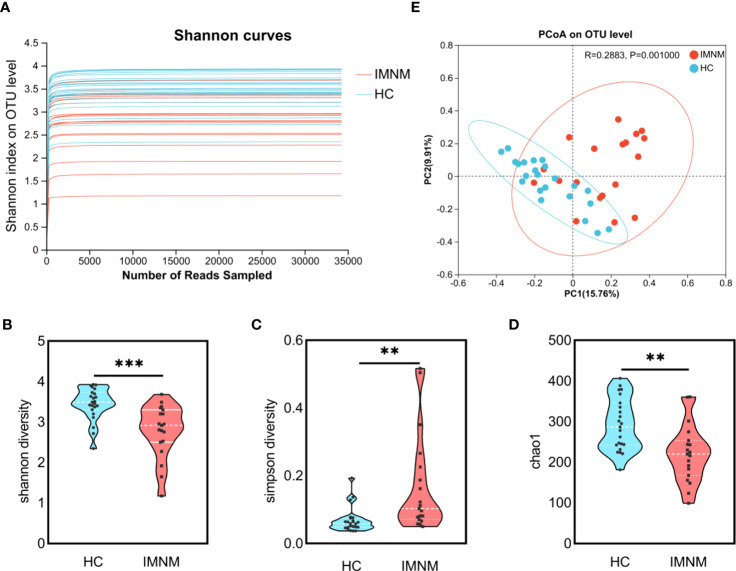
Comparative analysis of diversity and richness of the gut microbiota between HC and IMNM groups. **(A)** Rarefaction curves of all samples on the Shannon diversity index. **(B–D)** Shannon, Simpson, and Chao1 indexes of gut microbiota between HC and IMNM groups. **(E)** PCoA based on Bray-Curtis distances of gut microbiota between HC and IMNM groups. IMNM, immune-mediated necrotizing myopathy; HC, healthy control; PCoA, principal coordinates analysis. **p < 0.01 and ***p < 0.001. ns, no statistical difference.

Nineteen IMNM patients and 23 HCs were included in our research. Demographic characteristics, clinical features, and laboratory data of the two groups are shown in **Table 1**. The proportion of IMNM patients who used glucocorticoids was 94.74% (18/19), while the proportion of IMNM patients who used DMARDs was 57.89% (11/19). Additionally, IMNM was accessed by MITAX (18.26 ± 8.62) and MYOACT (20.68 ± 8.65) scores. The average age of IMNM and HC groups was 56.50 ± 7.72 and 50.35 ± 8.55 years, respectively. No significant difference was found between the groups (p = 0.383). The ratio of women to people in the IMNM group was 9/19 (47.37), and that in the healthy group was 11/23 (47.82). The gender distribution of the two groups also had no difference (p > 0.999). A total of 42 samples were used for 16S rDNA gene sequencing in our study. The rarefaction curves of all samples on the Shannon index reached a plateau, suggesting that the sequencing depth commonly covered all species and that the sequencing data had reached saturation, which indicates that the sequencing depth could reflect the community structure and diversity of the species to some extent (**Figure 1A**).

**Table 1:** Clinical manifestations and laboratory data of IMNMs and HCs.

Characteristics	HC (n=23)	IMNM (n=19)	*p*
Demographic data			
Age (years), mean±SD	50.35±8.55	56.50±7.72	*0.383*
Female sex, no./total no (%)	11/23 (47.82)	9/19 (47.37)	*>0.999*
BMI (kg/m^2^), mean±SD	/	21.35±3.35	/
Disease course (months), median (range)	/	18.25 (1, 108)	/
Clinical manifestation, no./total no (%)			
myasthenia	/	15/19 (78.95)	/
myalgia	/	10/19 (52.63)	/
amyotrophy	/	4/19 (21.05)	/
**MITAX score**	/	18.26±8.62	
**MYOACT score**	/	20.68±8.65	
**Laboratory data**	/		/
ANA (+), no./total no (%)	/	15/19 (78.95)	/
Anti-SSA (+), (no./total no (%))	/	5/19 (26.32)	/
Anti-Ro-52 (+), (no./total no (%))	/	6/19 (31.58)	/
HMGCR (+), (no./total no (%))	/	3/19 (15.79)	/
SRP (+), (no./total no (%))	/	9/19 (47.37)	/
ALT (IU/L), median(range)	/	112.47 (19, 334)	/
AST (IU/L) median(range)	/	106.16 (12, 302)	/
CK (IU/L), median(range)	/	2621.58 (38, 9453)	/
LDH (IU/L), median(range)	/	637.58 (127, 1484)	/
HBDH (IU/L), median(range)	/	500.95 (91, 1199)	/
MYO (ng/mL), median(range)	/	1045.817 (21, 3000)	/
CKMB (ng/mL), median(range)	/	134.11 (1.75, 300)	/
cTnT (ng/L), median(range)	/	688.77 (9.9, 2131)	/
ESR (%), median(range)	/	31 (2, 113)	/
CRP (mg/L), median(range)	/	8.74 (1, 47.4)	/
Occurrence of complication, no./total no (%)			
ILD (%)	/	8/19 (42.11)	/
diabetes mellitus II	/	2/19 (10.53)	/
hypertension	/	3/19 (15.79)	/
hyperlipemia	/	7/19 (36.84)	/
fatty liver	/	3/19 (15.79)	/
hyperuricemia	/	1/19 (5.26)	/
medication situation			
glucocorticoid	/	18/19 (94.74)	/
DMARDs	/	11/19 (57.89)	

IMNM, immune-mediated necrotizing myopathy; HC, healthy control; SD, standard deviation; BMI, body mass index; ANA, antinuclear antibody; SSA, Anti-SSA antibody; Anti-Ro-52, Anti-Ro-52 antibody; SRP, signal recognition particle; HMGCR, 3-hydroxy-3-methylglutaryl-coenzyme A reductase; AST, aspartate aminotransferase, ALT, alanine aminotransferase; LDH, lactate dehydrogenase, HBDH, hydroxybutyrate dehydrogenase; CK, creatine kinase; MYO, myoglobin; CKMB, creatine kinase muscle and brain isoenzyme; ; cTnT, cardiac troponin T; ESA, erythrocyte sedimentation rate; CRP, C-reactive protein; ILD, interstitial lung disease; DMARDs, disease-modifying anti-rheumatic drugs; MITAX, myositis intention to treat index; MYOACT, myositis disease activity assessment visual analogue scale. The meaning of the symbols "/" was "untested" ; the meaning of the symbols "(+)" was "positive".

Gut microbiota diversity and richness of HC and IMNM groups were evaluated by Shannon, Simpson, and Chao1 indexes based on the OTU species and abundance. Community diversity indexes, including the Shannon index (p = 0.0002) (**Figure 1B**) and Simpson index (p = 0.002) (**Figure 1C**), were significantly different between HC and IMNM groups. Additionally, the Chao1 index (p = 0.001) indicated that the OTU richness was obviously different between the two groups (**Figure 1D**). Gut microbiota diversity and richness of IMNM patients were lower than those of healthy people.

Beta-diversity was illustrated by PCoA based on Bray-Curtis distances to identify whether the overall structure of the gut microbiota in the HC group differed from that in the IMNM group. We found that beta-diversity plots could differentiate the gut microbiota of the two groups (ANOSIM test; R = 0.288, p = 0.001) (**Figure 1E**).

The number of common OTU units in IMNM and HC groups was 576, the number of unique OTU units in the HC group was 188, and the number of unique OTU units in the IMNM group was 394, showing a statistically significant difference (p < 0.001) (**Supplementary Figure 2**).

### 3.2 Differential distribution of gut microbiota phyla and genera in IMNM patients compared to HCs

**Figure 2:**
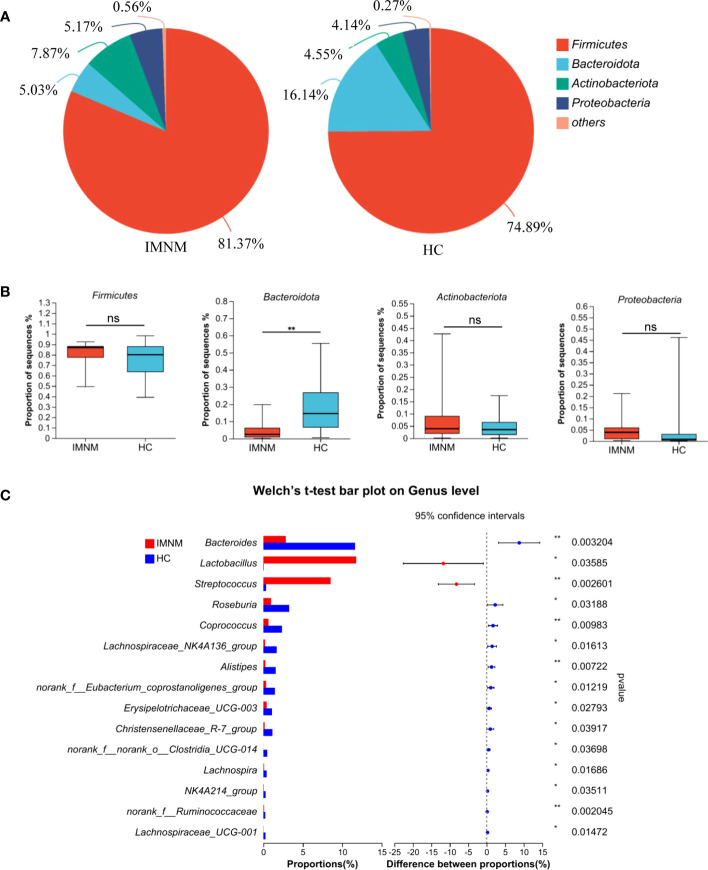
Differentially abundant taxa at phylum and genus levels between HC and IMNM groups. **(A)** Pie plot of bacterial community abundance at the phylum level in IMNM and HC groups. **(B)** The relative abundance of the dominant phyla, including *Firmicutes, Bacteroidota, Actinobacteriota*, and *Proteobacteria*, between the two groups. **(C)** Differential genera between the two groups, with only the top 15 genera being shown. IMNM, immune-mediated necrotizing myopathy; HC, healthy control. *p < 0.05 and **p < 0.01.

We analyzed microbial richness at the phylum level. The pie charts revealed that the dominant phyla across all subjects were Firmicutes, Bacteroidota, Actinobacteriota, and Proteobacteria, whose relative abundance together accounted for more than 99% of bacterial sequences (**Figure 2A**). Among these four dominant bacteria, the relative abundance of Bacteroidota distinctly decreased from the HC group to the IMNM group (p = 0.003), while alterations in the others were not statistically significant (**Figure 2B**).

**Figure 3:**
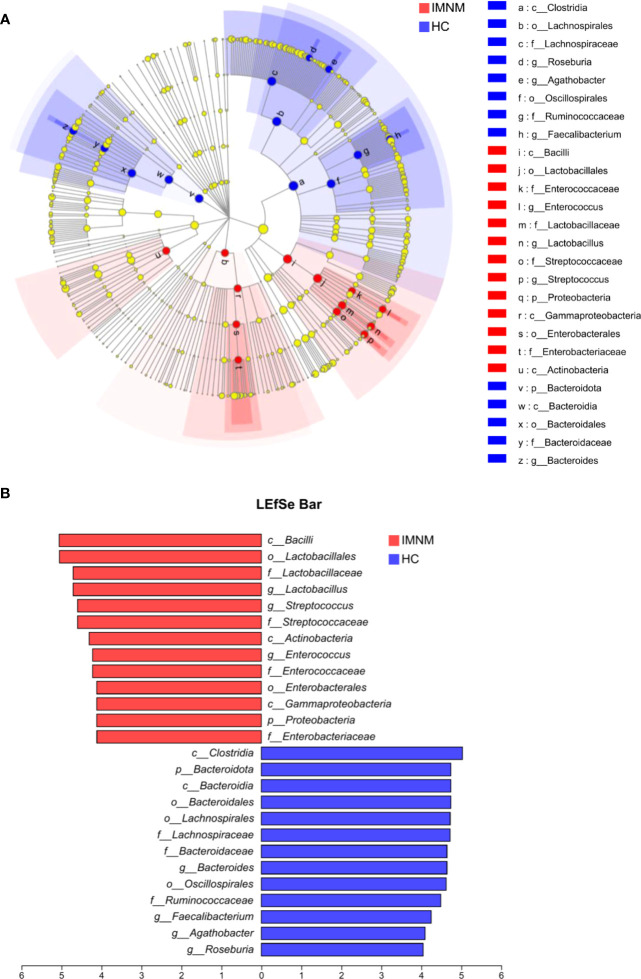
LEfSe analysis to identify differentially abundant taxa in IMNM and HC groups. **(A)** The phylogenetic tree in cladogram format of LEfSe analysis. **(B)** The greatest differences from phylum to genus levels between IMNM and HC groups. Linear discriminant analysis, LDA score (log10), LDA score > 4. LDA, linear discriminant analysis; LEfSe, linear discriminant analysis (LDA) effect size; IMNM, immune-mediated necrotizing myopathy; HC, healthy control.

Then, we focused on specific differences in microbiota composition at the genus level between the two groups. The considerable difference in the distribution of gut microbiota at the genus level in the two groups was shown by the genus bar plot, and the top dominant genus in the HC group was *Bacteroides*, while that in the IMNM group was *Lactobacillus* (**Supplementary Figure 3A**). Furthermore, we applied Welch’s t test to compare differences in genera between the two groups (**Figure 2C**). In IMNM patients, the relative abundances of *Bacteroides*, *Roseburia*, and *Coprococcus* were decreased compared to those in the HC group (p < 0.05) (**Supplementary Figure 3B**), while the relative abundances of *Lactobacillus* and *Streptococcus* were relatively increased (p < 0.05) (**Supplementary Figure 3C**). The above-mentioned findings were further verified by LefSe, as shown in the cladogram, and the contributory discriminate taxa with LDA scores > 4 were plotted for each group (**Figures 3A, B**).

### 3.3 Gut microbiota dysbiosis was related to clinical indicators in IMNM patients

**Figure 4:**
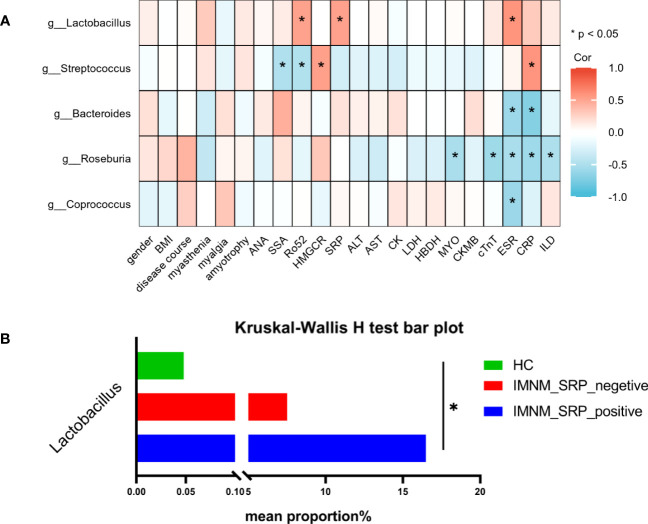
The correlation between gut microbiota and clinical indicators in IMNM patients. **(A)** Heatmap of Spearman correlation analysis between the top 5 differential microbial genera and 22 clinical indicators in IMNM patients. **(B)** The abundance of *Lactobacillus* based on subgroup analysis according to the level of anti-SRP antibodies. IMNM, immune-mediated necrotizing myopathy; BMI, body mass index; ANA, antinuclear antibody; SSA, anti-SSA antibody; Anti-Ro-52, anti-Ro-52 antibody; SRP, signal recognition particle; HMGCR, 3-hydroxy-3-methylglutaryl-coenzyme A reductase; AST, aspartate aminotransferase, ALT, alanine aminotransferase; LDH, lactate dehydrogenase, HBDH, hydroxybutyrate dehydrogenase; CK, creatine kinase; MYO, myoglobin; CKMB, creatine kinase muscle and brain isoenzyme; cTnT, cardiac troponin T; ESA, erythrocyte sedimentation rate; CRP, C-reactive protein; ILD, interstitial lung disease; Cor, correlation. *p < 0.05.

We assessed the association between gut microbiota changes and clinical indicators. Spearman correlation analysis was performed to analyze the correlation between the top five differential genera, including *Lactobacillus*, *Bacteroides*, *Streptococcus*, *Roseburia*, and *Coprococcus*, and 22 clinical indicators with (1) sex, BMI, and disease course of IMNM; (2) myositis antibodies, including anti-SRP antibodies, anti-HMGCR antibodies, ANA, SSA, and Ro52; (3) clinical symptoms and signs, including myasthenia, myalgia, and amyotrophy; (4) disease activity parameters, including AST, ALT, CK, LDH, HBDH, MYO, CKMB, cTnT, ESR, CRP C3 and C4; and (5) involvement of ILD (**Figure 4**).

In IMNM patients, the relative abundance of *Lactobacillus* was positively correlated with the levels of anti-SRP and anti-Ro52 antibodies and ESR, while *Streptococcus* was positively correlated with anti-HMGCR antibodies and CRP. Both genera were significantly increased in IMNM patients. Furthermore, *Roseburia*, which was decreased in IMNM patients, was negatively correlated with the serum levels of MYO, cTnT, ESR, and CRP and the occurrence of ILD, suggesting that *Roseburia* was associated with disease activity and lung involvement. *Bacteroides* was negatively correlated with ESR and CRP, while *Coprococcus* was negatively correlated with ESR, and both genera were significantly decreased in IMNM patients. The findings revealed no significant correlation between the five differential genera and the serum levels of C3 and C4 in 15 IMNM patients (Four IMNM patients did not undergo testing for C3 and C4 levels). (**Supplementary Figure 4**).

*Lactobacillus* is one of the probiotics present in the human intestine that improves the intestinal microenvironment, regulates immunity, and promotes nutrient absorption (Chang et al., 2019). *Lactobacillus* is significantly decreased in SLE (Mu et al., 2017) and IIM patients (Zhufeng et al., 2022). Our results demonstrated that the abundance of *Lactobacillus* increased and was positively correlated with the level of anti-SRP antibodies, which play an important role in IMNM patients. Therefore, we performed the subgroup analysis according to the expression of anti-SRP antibodies. All participants were divided into three groups: healthy individuals (HCs), SRP-negative IMNM patients (IMNM_SRP_negative), and SRP-positive IMNM patients (IMNM_SRP_positive). There was a significant statistical difference among the three groups, and the abundance of *Lactobacillus* was the highest in SRP-positive IMNM patients, the second in SRP-negative patients, and the lowest in HCs (**Figure 4B**).

### 3.4 Interactions among differential genera in IMNM patients

**Figure 5:**
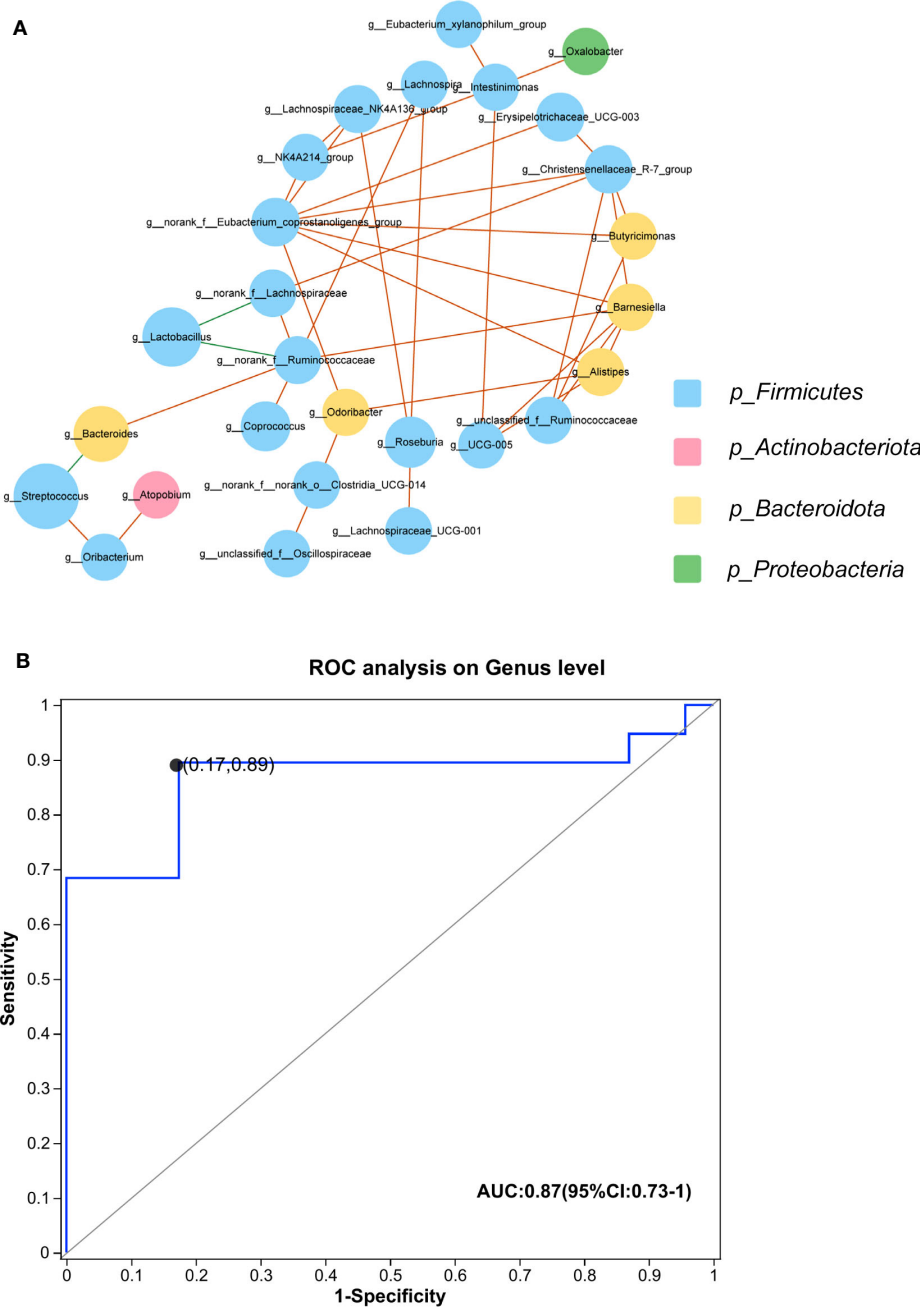
**(A)** Plots of co-abundance and co-exclusion association networks between differentially abundant genera. Each node represents one genus. The node size is proportional to the mean relative abundance of the genus in all samples. Node color indicates the phylum to which it belongs. Lines between nodes show positive correlations (solid orange lines) or negative correlations (solid green lines). **(B)** The built ROC curve based on the top 5 differential genera yielded an AUC of 0.87 (CI, 73%–100%). IMNM, immune-mediated necrotizing myopathy; AUC, area under the curve; ROC, receiver operator characteristic; CI, confidence interval.

We established a correlation network to explore co-abundance and co-exclusion interactions between 37 differential genera, as shown in **Figure 5A**. The correlated genera were from four phyla, including Firmicutes, Bacteroidota, Actinobacteriota, and Proteobacteria, and the majority of genera belonged to *Firmicutes*. *Lactobacillus* was negatively associated with *norank_f_Lachnospiraceae* and *norank_f_Ruminococcaceae*. Additionally, *Bacteroides* and Streptococcus were negatively correlated. The other correlations within the community were positive.

### 3.5 Prediction model of the gut microbiota biomarker profile in IMNM patients

We constructed a prediction model using the top five differential genera, including *Lactobacillus*, *Bacteroides*, *Streptococcus*, *Roseburia*, and *Coprococcus*, to distinguish IMNM patients from healthy individuals. The efficacy of these differential genera in discriminating between IMNM patients and healthy people was set up by using a ROC curve. The AUC was 87%, and 95% confidence interval (CI) was 73%–100% (**Figure 5B**).

## 4 Discussion

To our knowledge, this is the first study to demonstrate compositional alterations in the gut microbiota in IMNM patients. We found that the gut microbiota of IMNM patients was significantly different from that of healthy individuals and that the abundance and evenness of the gut microbiota in IMNM patients were decreased. Additionally, some fecal taxa changes were associated with clinical disease indicators in patients with IMNM. Furthermore, bacterial biomarker prediction models were successfully built to distinguish IMNM patients from healthy individuals. The above-presented results suggested that changes in gut microbiota might contribute to the pathogenesis of disease development in IMNM patients.

The results of the analysis of gut microbiota diversity showed that the gut microbiota in the IMNM group was significantly different from that of HCs. The abundance and evenness of gut microbiota in IMNM patients were significantly decreased. The number of OTUs in the two groups shown by the Venn diagram further suggested a significant reduction in gut microbiota diversity in IMNM patients. This finding was consistent with the alteration of gut microbiota diversity between IIM and healthy people reported in relevant studies (Luo et al., 2022; Zhufeng et al., 2022) and the results of related studies in other autoimmune diseases (Chen et al., 2021; Vieira et al., 2021).

The composition of the gut microbiota in IMNM patients changed significantly compared with that of healthy individuals. Healthy gut microbiota is predominantly composed of phyla *Firmicutes* and *Bacteroidetes*, which play vital roles in metabolic functions, maintenance of the intestinal barrier, and immune system function (Jandhyala et al., 2015). In our research, *Firmicutes* had the highest abundance in the two groups, followed by phylum *Bacteroidetes*. Additionally, differential genera were found mostly in Firmicutes (*Lactobacillus*, *Streptococcus*, *Roseburia*, and *Coprococcus*) and Bacteroidota (*Bacteroides*). Interestingly, the levels of myositis-associated autoantibodies in IMNM patients were associated with differential genera, including *Lactobacillus* and *Streptococcus*. *Lactobacillus* increased in the gut microbiota of IMNM patients and was positively correlated with myositis-specific antibodies SRP and myositis-associated autoantibodies Ro-52. *Streptococcus* increased and was positively correlated with myositis-specific antibodies HMGCR but negatively correlated with myositis-associated autoantibodies anti-Ro-52 antibody. We performed the subgroup analysis according to the expression of anti-SRP antibodies to further study the abnormal elevation of *Lactobacillus.* The result showed that the abundance of *Lactobacillus* was the highest in SRP-positive IMNM patients, the second in SRP-negative patients, and the lowest in healthy individuals. However, *Lactobacillus* is a probiotic (Chang et al., 2019). Wilck et al. (Wilck et al., 2017) reported that *Lactobacillus murinus* prevented salt-induced aggravation of actively induced experimental autoimmune encephalomyelitis and salt-sensitive hypertension by modulating T17 cells. Mu et al. (Mu et al., 2017) found that in a classical model of lupus nephritis, MRL/lpr, *Lactobacillales* were decreased in the gut microbiota, and increasing *Lactobacillales* could improve the renal function of these mice and prolonged their survival. Kang et al. (Kang et al., 2022) found that *Lactobacillus acidophilus* maintains intestinal barrier integrity, reduces metabolic endotoxemia, and inhibits the TLR4/NF-κB signaling pathway. Zegarra-Ruiz et al. (Zegarra-Ruiz et al., 2019) revealed the change of L. *Lactobacillus reuteri* in SLE mice and *Lactobacillus* enrichment in the feces of a subset of SLE patients. Additionally, they found that *Lactobacill*us reuteri colonization worsened autoimmune manifestations by notably increasing plasmacytoid dendritic cells (pDCs) and interferon signaling (Kawanabe-Matsuda et al., 2022). Thus, different species of *Lactobacillus* play various roles in different diseases, including positive and negative effects, which might be the reason that our result was inconsistent with reports that *Lactobacillus* decreases in other autoimmune diseases. However, the causality and relevant profound mechanism between *Lactobacillus* and anti-SRP antibodies are unclear and require further research.

In this study, inflammatory markers ESR and CRP were associated with the differential genera in IMNM patients. The level of ESR increased, accompanied by an increase in *Lactobacillus* and a decline in *Bacteroides*, *Roseburia*, and *Coprococcus*. Additionally, the serum concentration of CRP increased along with an increase in *Streptococcus* and a decrease in *Bacteroides* and *Roseburia*.*Bacteroides* decreased in IMNM patients compared with that in HCs and negatively correlated with the levels of ESR and CRP, which is consistent with the reported results in IIM patients (Luo et al., 2022). *Bacteroides* play an important role in metabolizing dietary and host glycans by polysaccharide utilization (Charbonneau et al., 2016) and synthesize conjugated linoleic acid, which is known to be antidiabetic, antiatherogenic, antiobesogenic, hypolipidemic, and immunomodulatory (Baddini Feitoza et al., 2009; Fukiya et al., 2009). Typical butyrate-producing bacteria *Roseburia* was reduced in inflammatory bowel disease and negatively correlated with CRP, contributing to the pathogenesis of ulcerative colitis and Crohn’s disease (Jiang et al., 2016), which is consistent with our result demonstrated above. Butyrate, as a presence of the bacterial metabolite, could dramatically inhibit the differentiation of monocytes into macrophages and suppresses the secretion of pro-inflammatory cytokines such as TNF-α and IL-6 by macrophages (Chen et al., 2018; Schulthess et al., 2019). In our study, the typical butyrate producing bacteria *Roseburia* was reduced and negatively correlated with the serum level of CRP, which is consistent with the study of inflammatory bowel disease (Jiang et al., 2016). The activation of the complement system is a significant pathological process in IMNM (Allenbach et al., 2020). Nevertheless, it should be noted that the observed correlation between the five he five top differential genera and the levels of C3 and C4 in our study might be attributed to the limited size of the sample population, implying that a comprehensive investigation with a larger sample size is warranted to further explore.

ILD is a kind of organ involvement in IMNM patients and affects their prognosis (Ge et al., 2022). In this study, *Roseburia*, which declined significantly in the gut microbiota of IMNM patients, was negatively correlated with the occurrence of ILD. *Roseburia* is also decreased in some lung diseases, such as non-small cell lung cancer, respiratory infections, and asthma (Gui et al., 2020; Alcazar et al., 2022). *Roseburia* is a typical butyrate-producing bacterium (Jiang et al., 2016). Kabel et al. reported that butyrate could alleviate BLM-induced pulmonary fibrosis in rats by inhibiting nuclear factor kappa-B (Kabel et al., 2016). Park et al. reported that butyrate might exhibit indirect and direct anti-fibrogenic action on fibroblasts by regulating macrophage differentiation and inhibition of histone deacetylase 3, ameliorating skin and lung fibrosis in a mouse model of SSc (Park et al., 2021). These results suggested that *Roseburia* might be a protective factor against ILD by producing butyrate in IMNM patients. Myasthenia and myalgia are typical symptoms of IMNM patients and are accompanied by increased muscle enzymes, including AST, ALT, CK, LDH, HBDH, MYO, CKMB, and cTnT (Allenbach et al., 2020; Merlonghi et al., 2022). *Roseburia* was negatively correlated with serum levels of MYO and cTnT. *Roseburia* was also found to be decreased in sarcopenia patients (Ticinesi et al., 2020; Wu et al., 2020; Kang et al., 2021). Butyrate absorbed into the systemic circulation could regulate protein synthesis in skeletal muscle and negatively affect glucose metabolism in skeletal muscle cells (Canfora et al., 2015; Nay et al., 2019). Walsh et al. (Walsh et al., 2015) reported that butyrate improves metabolism and reduces muscle atrophy during aging by regulating protein and glucose metabolism and antioxidative activity. These results suggest that *Roseburia* might play a protective role in organ damage (lungs and muscles) by producing butyrate.

However, there were some limitations to our study. First, the number of participants with IMNMs included was inadequate because of the rarity of the disease, and all participants were recruited from a single hospital, which might not fully represent the diversity of IMNM patients and HCs. A multi-center study with more participants would enhance the external validity. Second, most IMNM patients were receiving glucocorticoids and DMARDs, which might affect the gut microbiota. Third, the collection of samples was not systematic. Fourth, IMNM patients were probably hospitalized more frequently than HCs, which might have influenced the gut microbiota. Furthermore, the results from 16S rDNA gene sequencing did not provide species-level analysis, making it impossible to perform a more detailed analysis.

## 5 Conclusion

Taken together, we demonstrate that IMNM is characterized by gut microbial dysbiosis with an abnormal elevation of *Lactobacillus*, some of which is associated with myositis antibodies and inflammatory markers, suggesting that gut microbiota dysbiosis occurs in IMNM patients and is correlated with systemic autoimmune features. However, research on IMNM and gut microbiota is still in its infancy and requires well-designed prospective human studies and animal flora transplantation experiments to warrant further insights into possible causal relationships.

## Data availability statement

The fastq files were deposited into the NCBI Sequence Read Archive (SRA) database (Accession Number: PRJNA937002).

## Ethics statement

The study was approved by the ethical committee of West China Hospital of Sichuan University (No. 695 in 2020) and complied with the Declaration of Helsinki. The study did not involve animal studies, that no ethical approval is required.

## Author contributions

YL and YBL conceived, designed and guided the study. XL, YHL, LC, YW, TW, JW, DH, ZL and CT collected the clinical samples. XL, YHL, and LC analyzed the data. All authors drafted and revised the manuscript. XL and YHL contributed equally to this work. All authors contributed to the article and approved the submitted version.
