# Supplementary material for: Gut microbiota dysbiosis characterized by abnormal elevation of Lactobacillus in patients with immune-mediated necrotizing myopathy
Source: Front Cell Infect Microbiol. 2023 Aug 25;13:1243512. doi: 10.3389/fcimb.2023.1243512 (PMC10486907; doi:10.3389/fcimb.2023.1243512)
Supplement: Supplementary file 1 [file DataSheet_1.docx]

Supplementary Material


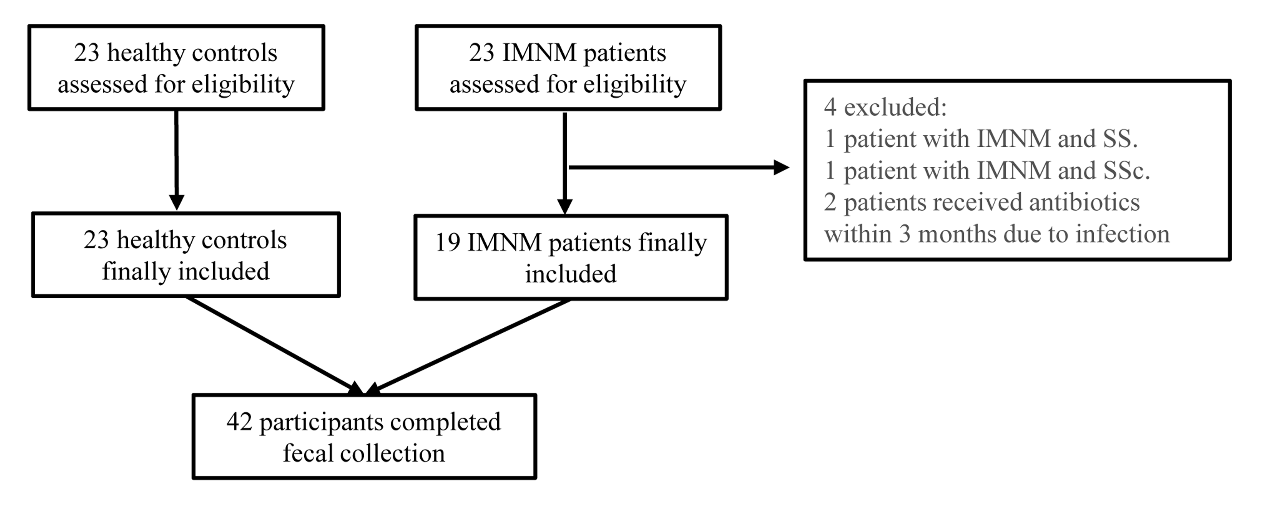


**Supplementary Figure 1** The flow chart of participants inclusion. Abbreviation: IMNM: immune-mediated necrotizing myopathy; SS: Sjögren’s syndrome; SSc: systemic sclerosis.


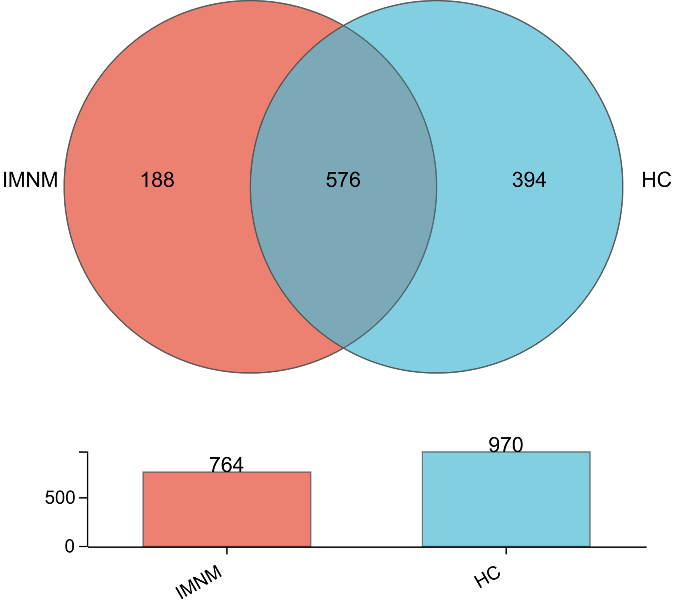


Supplementary Figure 2 Venn diagram of IMNM and HC groups at the OTU level. Abbreviation: IMNM: immune-mediated necrotizing myopathy; HC: healthy control; OUTs: operational taxonomic units.


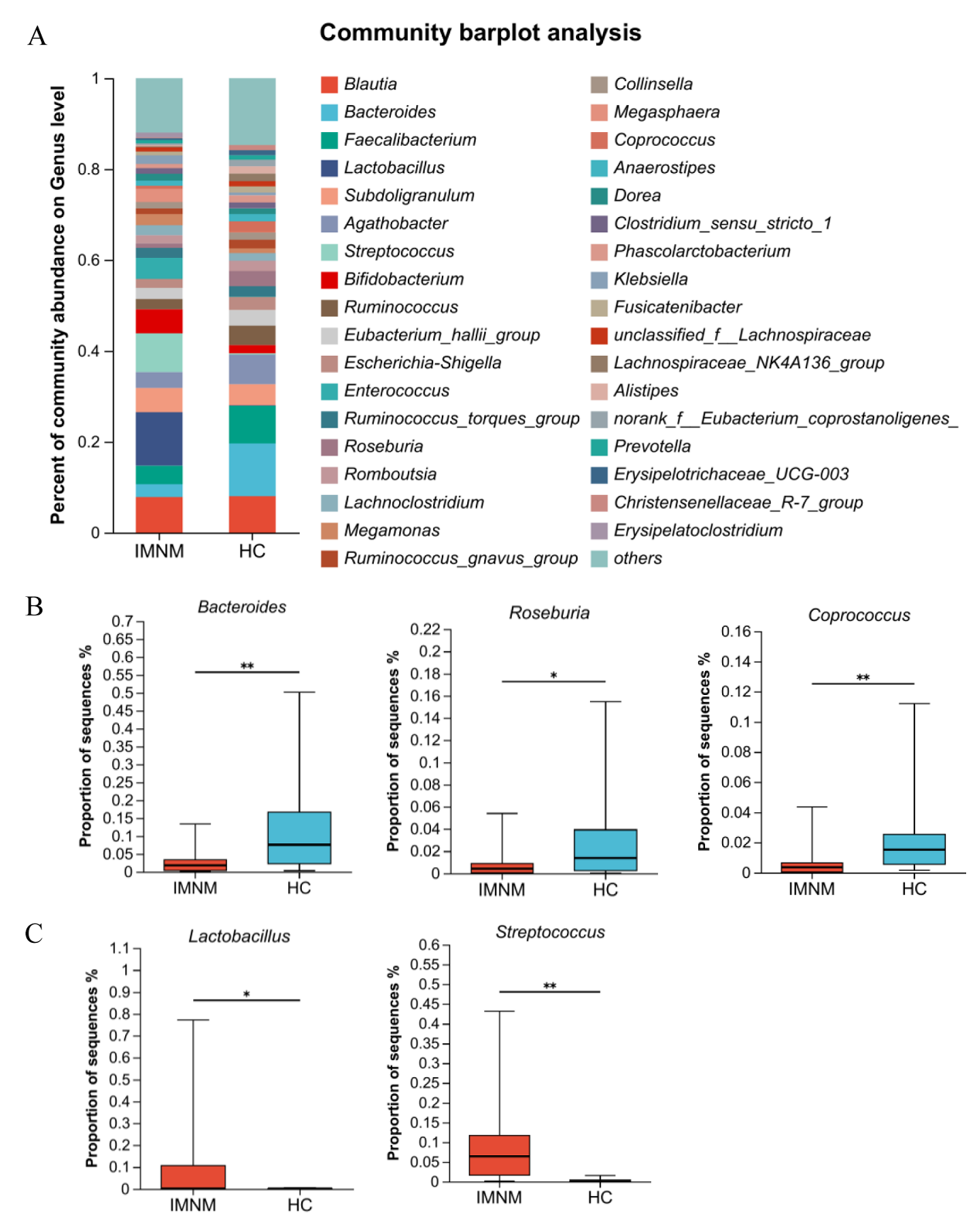


**Supplementary Figure 3**. Supplementary Figure 3. Composition and abundance distribution of gut microbiota in HC and IMNM groups at the genus level. (A) Bar plot of the bacteria in HC and IMNM groups at the genus level. (B–C) The relative abundance of differential genera in the top 15 genera, including *Bacteroides*, *Lactobacillus*, Streptococcus, *Roseburia*, and *Coprococcus*, between the two groups. Abbreviation: IMNM: immune-mediated necrotizing myopathy; HC: healthy control. *p < 0.05 and **p < 0.01.


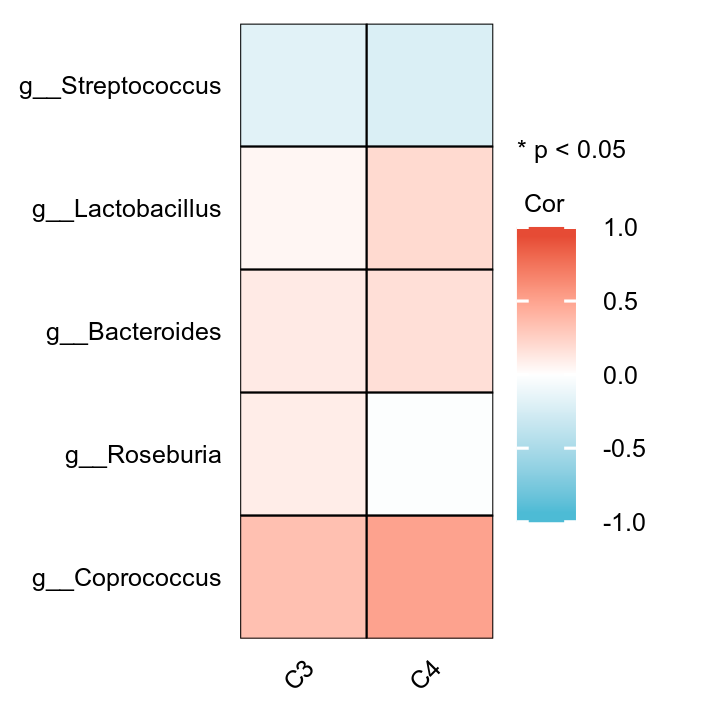


**Supplementary Figure 4.** The correlation between the top 5 differential microbial genera and complement (C3 and C4) in IMNM patients(n=15). Abbreviations: IMNM: immune-mediated necrotizing myopathy; Cor: correlation. *p < 0.05.
